# 603. Access to Pediatric Burn Care in the US: Where to Place Additional Verified Burn Centers

**DOI:** 10.1093/jbcr/irag033.407

**Published:** 2026-04-06

**Authors:** Meg Quint, Matthew Uzelac, Clifford C Sheckter

**Affiliations:** Stanford University School of Medicine, Stanford, CA; Stanford University School of Medicine, Stanford, CA; Stanford University School of Medicine, Stanford, CA

## Abstract

**Introduction:**

Pediatric burn patients require specialized care to manage the nuances in physiology and deliver tailored surgical care. The American Burn Association (ABA) champions treatment at pediatric specific verified centers, but access to verified pediatric centers remains geographically limited. Using simulations, we aimed to identify geographic locations in the US where pediatric burn care is limited, and where pediatric verified centers could be placed to improve access.

**Methods:**

We used the 2017-2019 and 2022 National Inpatient Sample to identify pediatric burn hospitalizations (ICD-10: T20-T32, patients <18) across the nine U.S. Census divisions. A division’s maximum capacity was defined as two standard deviations above their average annual incidence of pediatric burns. We used Monte Carlo simulations to model three triage scenarios, routing pediatric burns to: 1) any verified burn center, 2) pediatric or combined (i.e., adult and pediatric) centers, and 3) pediatric or combined centers with additional pediatric centers to restore the number of unaccommodated patients per zip code tabulation area per year to those in scenario one. Candidate locations for new centers were selected using population densities and proximities to existing centers. After candidate cities were identified, we determined if there are current adult ABA-verified centers or non-verified pediatric centers that could feasibly seek ABA pediatric verification.

**Results:**

Routing all pediatric patients to pediatric or combined centers only (scenario 2) significantly increased overflow rates, particularly in South Atlantic, West South Central, and East North Central divisions. The placement of 20 additional pediatric centers reduced these overflow rates to those in the baseline scenario. Of these, each demonstrated considerable catchment populations (median: 7.16 million; IQR: 4.51-14.1 million) and average travel distance reductions (median: 94 miles; IQR: 24-180 miles). Of the 20 identified cities for additional centers, 15 (75%) had current burn programs (any verification status) that could feasibly seek pediatric verification. Of these 15, 5 (33%) are ABA-verified adult centers, 2 (13%) are pediatric non-ABA-verified centers. Thirteen (86%) have a pediatric ICU in their available hospital system.

Fig. 1A) demonstrates triage scenario one. Fig. 1B) demonstrates triage scenario three. Color shows average number of unaccommodated patients annually in each scenario.

**Conclusions:**

Strategic placement of additional pediatric ABA-verified burn centers would allow for additional pediatric patients to receive specific care without compromising system overload and while supporting reduced transport distance.

**Applicability of Research to Practice:**

These findings offer an evidence-based approach guiding strategic expansion of pediatric care to improve access and outcomes.

**Funding for the study:**

N/A.

